# *Trichoderma* Biodiversity of Agricultural Fields in East China Reveals a Gradient Distribution of Species

**DOI:** 10.1371/journal.pone.0160613

**Published:** 2016-08-02

**Authors:** Yuan Jiang, Jin-Liang Wang, Jing Chen, Li-Juan Mao, Xiao-Xiao Feng, Chu-Long Zhang, Fu-Cheng Lin

**Affiliations:** 1 State Key Laboratory of Rice Biology, Institute of Biotechnology, Zhejiang University, Hangzhou, Zhejiang, China; 2 Analysis Center of Agrobiology and Environmental Science, Zhejiang University, Hangzhou, Zhejiang, China; Friedrich Schiller University, GERMANY

## Abstract

We surveyed the *Trichoderma* (Hypocreales, Ascomycota) biodiversity in agricultural fields in four major agricultural provinces of East China. *Trichoderma* strains were identified based on molecular approaches and morphological characteristics. In three sampled seasons (spring, summer and autumn), 2078 strains were isolated and identified to 17 known species: *T*. *harzianum* (429 isolates), *T*. *asperellum* (425), *T*. *hamatum* (397), *T*. *virens* (340), *T*. *koningiopsis* (248), *T*. *brevicompactum* (73), *T*. *atroviride* (73), *T*. *fertile* (26), *T*. *longibrachiatum* (22), *T*. *pleuroticola* (16), *T*. *erinaceum* (16), *T*. *oblongisporum* (2), *T*. *polysporum* (2), *T*. *spirale* (2), *T*. *capillare* (2), *T*. *velutinum* (2), and *T*. *saturnisporum* (1). *T*. *harzianum*, *T*. *asperellum*, *T*. *hamatum*, and *T*. *virens* were identified as the dominant species with dominance (*Y*) values of 0.057, 0.052, 0.048, and 0.039, respectively. The species amount, isolate numbers and the dominant species of *Trichoderma* varied between provinces. Zhejiang Province has shown the highest diversity, which was reflected in the highest species amount (14) and the highest Shannon–Wiener diversity index of *Trichoderma* haplotypes (1.46). We observed that relative frequencies of *T*. *hamatum* and *T*. *koningiopsis* under rice soil were higher than those under wheat and maize soil, indicating the preference of *Trichoderma* to different crops. Remarkable seasonal variation was shown, with summer exhibiting the highest biodiversity of the studied seasons. These results show that *Trichoderma* biodiversity in agricultural fields varies by region, crop, and season. Zhejiang Province (the southernmost province in the investigated area) had more *T*. *hamatum* than Shandong Province (the northernmost province), not only in isolate amounts but also in haplotype amounts. Furthermore, at haplotype level, only *T*. *hamatum* showed a gradient distribution from south to north in correspondence analysis among the four dominant species. The above results would contribute to the application of *Trichoderma* biocontrol strains.

## Introduction

Species in the fungal genus *Trichoderma* (Hypocreales, Ascomycota) possess rapid growth and high stress-tolerance. They are widely distributed and have a variety of biological activities. Some species, such as *T*. *harzianum* form the basis for commercial products applied for biological control of plant pathogenic fungi [1–3]. Some *Trichoderma* species have beneficial effects on plant growth and development [4, 5]. And some species, such as *T*. *reesei* have important industrial applications as producer of cellulolytic and hemicellulolytic enzymes [6].

New species of *Trichoderma* have recently been reported [7–9] and 254 named species were previously known [10, 11]. A broad array of secondary metabolites, many of which have novel bioactivity, are produced by *Trichoderma* species isolated from varied environments [12, 13]. Native *Trichoderma* strains in agricultural soils are likely to be more effective biocontrol agents, since they are already adapted to variable field conditions [14]. The prevalence of *Trichoderma* in different ecological niches contributes to the high diversity of genetic and metabolic variability [15]. Exploration of genetic diversity in this genus has been conducted in several regions [16–20].

*Trichoderma* has shown a considerably high genetic biodiversity not only at the species level but also at the under species level. For instance, *T*. *harzianum* and *T*. *asperellum* were represented by several genotypes or haplotypes [21, 22]. *Trichoderma* diversity has been explored in China, but only in a limited number of climatic regions (excluding major agricultural provinces) or only in a limited number of seasons in species level [21, 23]. We therefore focused our research on *Trichoderma* species and haplotype biodiversity within the major agricultural areas of China, investigated the influence of regional, seasonal and cropping variability and examined the species and amounts of *Trichoderma* to find the potential distribution pattern.

## Materials and Methods

No specific permissions were required for sampling locations/activities for native research institutes or researchers, and the studies did not involve endangered or protected species.

### Sampling

*Trichoderma* isolates in this study derived from soils of cultivated lands, orchards, and gardens in 20 regions of East China. The sampling sites were located in four major agricultural provinces (Anhui, Jiangsu, Shandong and Zhejiang). We collected 737 samples in total during May, August, and October of 2014 and 2015. The sampling sites at different months were the same, and the geographical coordinates were shown in S1 Table. Among these, 312 samples were from soil under rice cultivation, followed by maize (94) and wheat (75). Each sample contained about 200 g of soil from a depth of approximately 20 cm. Samples were placed into sterile polyethylene bags, transported to the laboratory, and stored at 4°C until isolation.

### Isolation and storage of Trichoderma strains

Rose Bengal Agar was the selective medium for *Trichoderma* and we used the soil dilution plating method. Putative *Trichoderma* colonies were purified by two rounds of subculturing on potato-dextrose agar (PDA). Pure cultures were suspended in cryopreservation liquid (15% v/v glycerol, 10 g/L glucose, 1 g/L yeast extract and 1 g/L casein hydrolysate) and stored at -70°C.

### DNA extraction, amplification, and sequencing

The mycelium of pure cultures was scraped directly from plates after 3 d growth on PDA at 25°C and used to extract DNA according to the method of [24]. To amplify internal transcribed spacer regions 1 and 2 (ITS1 and ITS2) of the ribosomal nucleotide sequence, a pair of general primers ITS5 (5'GGAAGTAAAAGTCGTAACAAGG3') and ITS4 (5'TCCTCCGCTTATTGATATGC3') were used. The PCR reaction was performed in a thermal cycler with the following parameters: 3 min initial denaturation at 94°C, followed by 30 cycles of 1 min denaturation at 94°C, 30 s primer annealing at 56°C, 30 s extension at 72°C, and a final extension period of 10 min at 72°C.

A 0.9-kb fragment of the 5' end of the translation elongation factor-1α (*tef*) gene (eEF1a1) containing three major introns was amplified using the primer pair *tef*71f (C AAA ATG GGT AAG GAG GAS AAG AC) and *tef*997R (CA GTA CCG GCR GCR ATR ATS AG) in the case where the ITS sequence did not provide unambiguous identification. The amplification program reference protocol was submitted by John Bissett (http://www.isth.info/isth/methods/method.php?method_id=9).

### Phylogenetic analysis

ITS sequences of all isolated *Trichoderma* strains were used to obtain haplotypes by DnaSP ver. 5.1 [25]. Sequences of known species including type strains and outgroup were downloaded in NCBI database. The alignment of type haplotypes and the known species was produced by Clustal W [26]. After the exclusion of leading and trailing gap regions, the nucleotide substitution model was selected using Jmodeltest [27]. A phylogenetic tree was obtained with the combination of Bayesian analysis and maximum parsimony analysis, which was performed by MrBayes v. 3.0b4 [28] and PAUP* 4.0 b10 [29], respectively.

### Statistical analysis

The dominance values were calculated using the following formula:

$$Y=\;\frac{n_{i}}{N}\times f_{i}\;,$$ (1)

where *N* is the total strain count, *n*_*i*_ is the count of genus (species) i, and *f*_*i*_ is the frequency that genus (species) *i* appears in the samples. The genus *i* is dominant while *Y* > 0.02. SPSS version 20.0 (IBM Corp, Armonk, NY, USA) was used for multiple correspondence analysis of sampling sites, based on the existence of the dominant species, respectively, and the CA bioplots were used for results analysis.

## Results

### Trichoderma isolation and Phylogenetic analyses

A total of 737 samples distributed across three seasons were collected from East China. All putative *Trichoderma* isolates were separated from Rose Bengal Agar to obtain a full-scale *Trichoderma* diversity profile. These isolates were preliminarily identified using morphological characters and ITS1 and 2 oligonucleotide barcode. In some cases, species pairs occurred because ITS1 and 2 sequences did not provide sufficient available species-specific diagnostic characters. If so, the translation elongation factor 1-alpha (*tef1-α*) gene was amplified for additional characters. A total of 2078 isolates were identified as *Trichoderma*. Among these, 2075 isolates were classified into 17 species: *T*. *harzianum* (429 isolates), *T*. *asperellum* (425), *T*. *hamatum* (397), *T*. *virens* (340), *T*. *koningiopsis* (248), *T*. *brevicompactum* (73), *T*. *atroviride* (73), *T*. *fertile* (26), *T*. *longibrachiatum* (22), *T*. *pleuroticola* (16), *T*. *erinaceum* (16), *T*. *oblongisporum* (2), *T*. *polysporum* (2), *T*. *spirale* (2), *T*. *capillare* (2), *T*. *velutinum* (2), and *T*. *saturnisporum* (1). The remaining three isolates were not identified at the species level.

**Fig 1:**
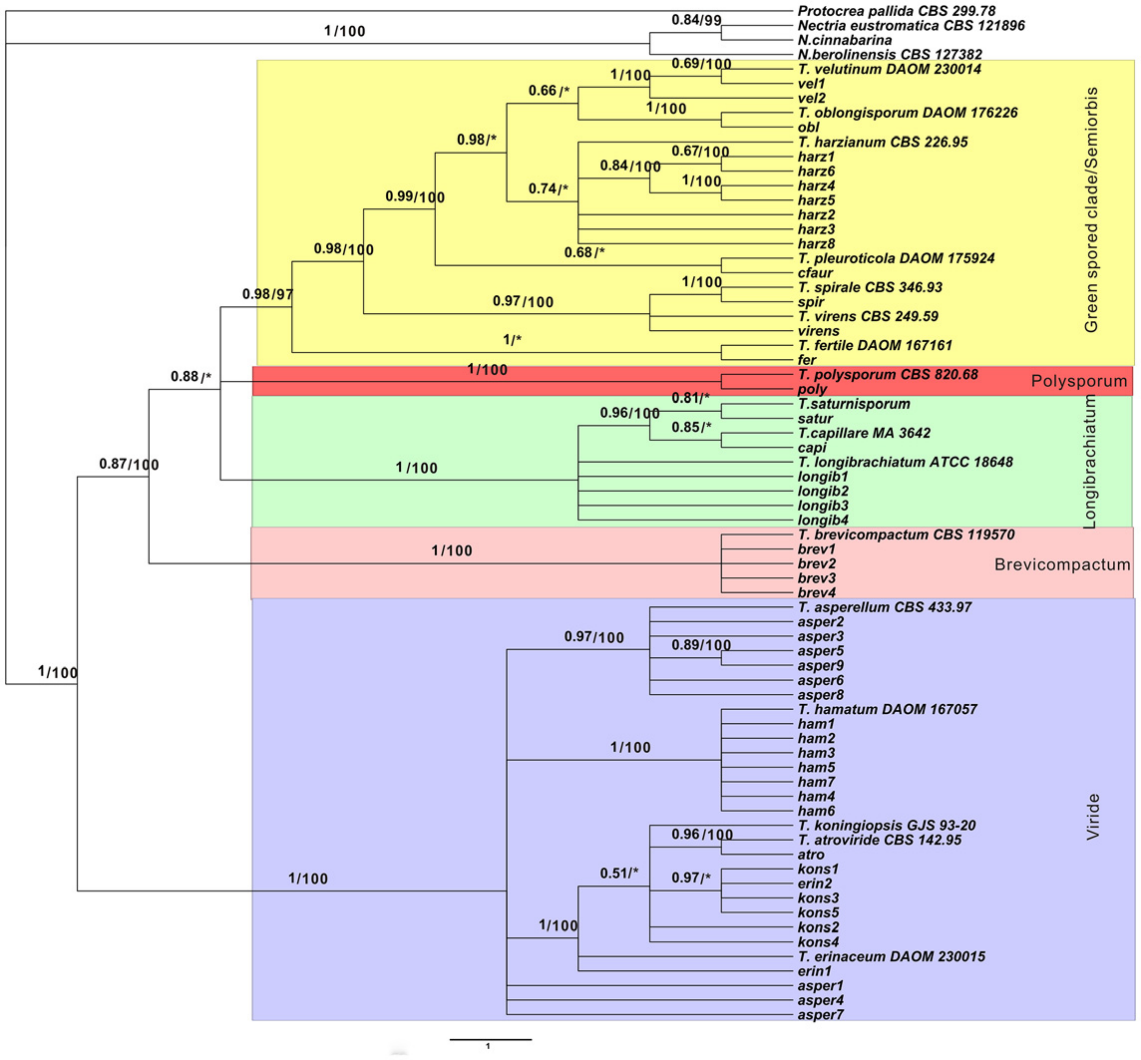
Phylogenetic tree inferred from ITS rDNA sequences. Bayesian posterior probabilities followed by maximum parsimony bootstrap values above 50 with 1000 replications are shown at the branch. Branches marked with an asterisk indicated that the branches were not present or have bootstrap values below 50 in the maximum parsimony tree. The bar represents 1.0 substitution per site.

To establish a phylogenetic tree, we first calculated haplotypes from 2078 ITS1 and 2 sequences. Finally, 8 haplotypes (Table 1) were subjected to parsimony and Bayesian analysis, which used *Protocrea pallida*, *Nectria eustromatica*, *N*. *berolinensis* and *N*. *cinnabarina* as the outgroup to identify the situation of the root. The result of this phylogenetic analysis is shown in Fig 1. The 48 haplotypes belonging to the 17 *Trichoderma* species were positioned into five clusters with strong bootstrap supports. The phylogenetic structure was consistent with previously established sections and clades in most cases [30, 31].

**Table 1:** Haplotypes (48) of *Trichoderma* identified in this study.

Haplotype Code	Species	Number of isolated strains	Representative strains	
			Code	GenBank Accession
asper1	*T*. *asperellum*	237	CTCCSJ-A-SD31000	KT314289
asper2	*T*. *asperellum*	173	CTCCSJ-A-SC31024	KT314294
asper3	*T*. *asperellum*	1	CTCCSJ-A-SC31096	KT314300
asper4	*T*. *asperellum*	1	CTCCSJ-A-SD31174	KT314302
asper5	*T*. *asperellum*	9	CTCCSJ-A-SC3140	KT314305
asper6	*T*. *asperellum*	1	CTCCSJ-A-GS31670	KT314308
asper7	*T*. *asperellum*	1	CTCCSJ-A-SC3254	KT314313
asper8	*T*. *asperellum*	1	CTCCSJ-A-XM337	KT314317
asper9	*T*. *asperellum*	1	CTCCSJ-A-SD3908	KT314330
atro	*T*. *atroviride*	73	CTCCSJ-A-SC350	KT314321
brev1	*T*. *brevicompactum*	67	CTCCSJ-A-SD31063	KT314299
brev2	*T*. *brevicompactum*	3	CTCCSJ-A-XM3257	KT314314
brev3	*T*. *brevicompactum*	2	CTCCSJ-A-GS3259	KT314315
brev4	*T*. *brevicompactum*	1	CTCCSJ-A-GS3847	KT314329
erin1	*T*. *erinaceum*	15	CTCCSJ-A-SC31615	KT314307
erin2	*T*. *erinaceum*	1	CTCCSJ-A-SC31443	KT314306
fer	*T*. *fertile*	26	CTCCSJ-A-YM3122	KT314304
ham1	*T*. *hamatum*	340	CTCCSJ-A-SD31002	KT314290
ham2	*T*. *hamatum*	6	CTCCSJ-A-SD31012	KT314293
ham3	*T*. *hamatum*	44	CTCCSJ-A-SD31214	KT314303
ham4	*T*. *hamatum*	1	CTCCSJ-A-SD3711	KT314327
ham5	*T*. *hamatum*	1	CTCCSJ-A-QT3177	KT314309
ham6	*T*. *hamatum*	1	CTCCSJ-A-SD3937	KT314332
ham7	*T*. *hamatum*	4	CTCCSJ-A-SD3550	KT314322
harz1	*T*. *harzianum*	235	CTCCSJ-A-SD31009	KT314292
harz2	*T*. *harzianum*	66	CTCCSJ-A-SD31031	KT314296
harz3	*T*. *harzianum*	21	CTCCSJ-A-XM31791	KU375457
harz4	*T*. *harzianum*	41	CTCCSJ-A-MH31042	KT314298
harz5	*T*. *harzianum*	26	CTCCSJ-A-SD3109	KT314301
harz6	*T*. *harzianum*	33	CTCCSJ-A-SD31059	KU375456
harz8	*T*. *harzianum*	7	CTCCSJ-A-SD3775	KU375462
satur	*T*. *saturnisporum*	1	CTCCSJ-A-SD3604	KT314324
kons1	*T*. *koningiopsis*	79	CTCCSJ-A-YC3100	KT314288
kons2	*T*. *koningiopsis*	164	CTCCSJ-A-SD31029	KT314295
kons3	*T*. *koningiopsis*	2	CTCCSJ-A-XM323	KT314311
kons4	*T*. *koningiopsis*	2	CTCCSJ-A-SD3388	KT314319
kons5	*T*. *koningiopsis*	1	CTCCSJ-A-SD3644	KT314325
longib1	*T*. *longibrachiatum*	18	CTCCSJ-A-SC3195	KT314310
longib2	*T*. *longibrachiatum*	1	CTCCSJ-A-SC378	KT314328
longib3	*T*. *longibrachiatum*	2	CTCCSJ-A-YM32425	KU375460
longib4	*T*. *longibrachiatum*	1	CTCCSJ-A-YM32819	KU375461
obl	*T*. *oblongisporum*	2	CTCCSJ-A-GS3677	KT314326
cfaur	*T*. *pleuroticola*	16	CTCCSJ-A-YM3383	KT314318
poly	*T*. *polysporum*	2	CTCCSJ-A-SD3918	KT314331
capi	*T*. *capillare*	1	CTCCSJ-A-SD3940	KT314333
spir	*T*. *spirale*	2	CTCCSJ-A-SC3230	KT314312
vel	*T*. *velutinum*	2	CTCCSJ-A-SD3579	KT314323
virens	*T*. *virens*	340	CTCCSJ-A-SD31006	KT314291

The first cluster includes clade Viride with *T*. *asperellum*, *T*. *hamatum*, *T*. *atroviride*, *T*. *erinaceum*, and *T*. *koningiopsis*. For the mutation at 115 and 371 nt (shown in S1 Fig) haplotypes of asper1, 4 and 7 do not cluster in the branch, which includes the other haplotypes and ex-type of *T*. *asperellum*. The four haplotypes of *T*. *brevicompactum* constitute the second cluster with Bayesian posterior probabilities of 1 and a maximum parsimony bootstrap value of 100. The third cluster (clade Longibrachiatum) includes *T*. *longibrachiatum*, *T*. *capillare*, and *T*. *saturnisporum*, and is strongly supported. The fourth cluster is clade polysporum, which only includes one species (*T*. *polysporum*). The remaining species were clustered with a Bayesian posterior probability of 0.98 and a maximum parsimony bootstrap value of 97. These species are distributed in two clades: green spored clade and semiorbis clade. The seven haplotypes of *T*. *harzianum* formed a moderately well-supported (0.74) clade.

### Species diversity

A total of 557 soil samples (isolation rate = 76%) produced *Trichoderma* isolates from 737 varied soil samples, and 2078 *Trichoderma* isolates (relative rate = 3%) were identified from 63426 fungal colonies. The dominance (*Y*) value was 0.025 (> 0.02), indicating that the genus *Trichoderma* was dominant in the soil samples. *T*. *harzianum*, *T*. *asperellum*, *T*. *hamatum*, and *T*. *virens* were the dominant species with dominance (*Y*) values of 0.057, 0.052, 0.047, and 0.039, respectively. The Shannon–Wiener diversity index of haplotypes was similar between the four provinces: 1.46 (Zhejiang), 1.43 (Jiangsu), 1.40 (Anhui), and 1.32 (Shandong). However, the proportion and composition of *Trichoderma* species varied between provinces.

**Fig 2:**
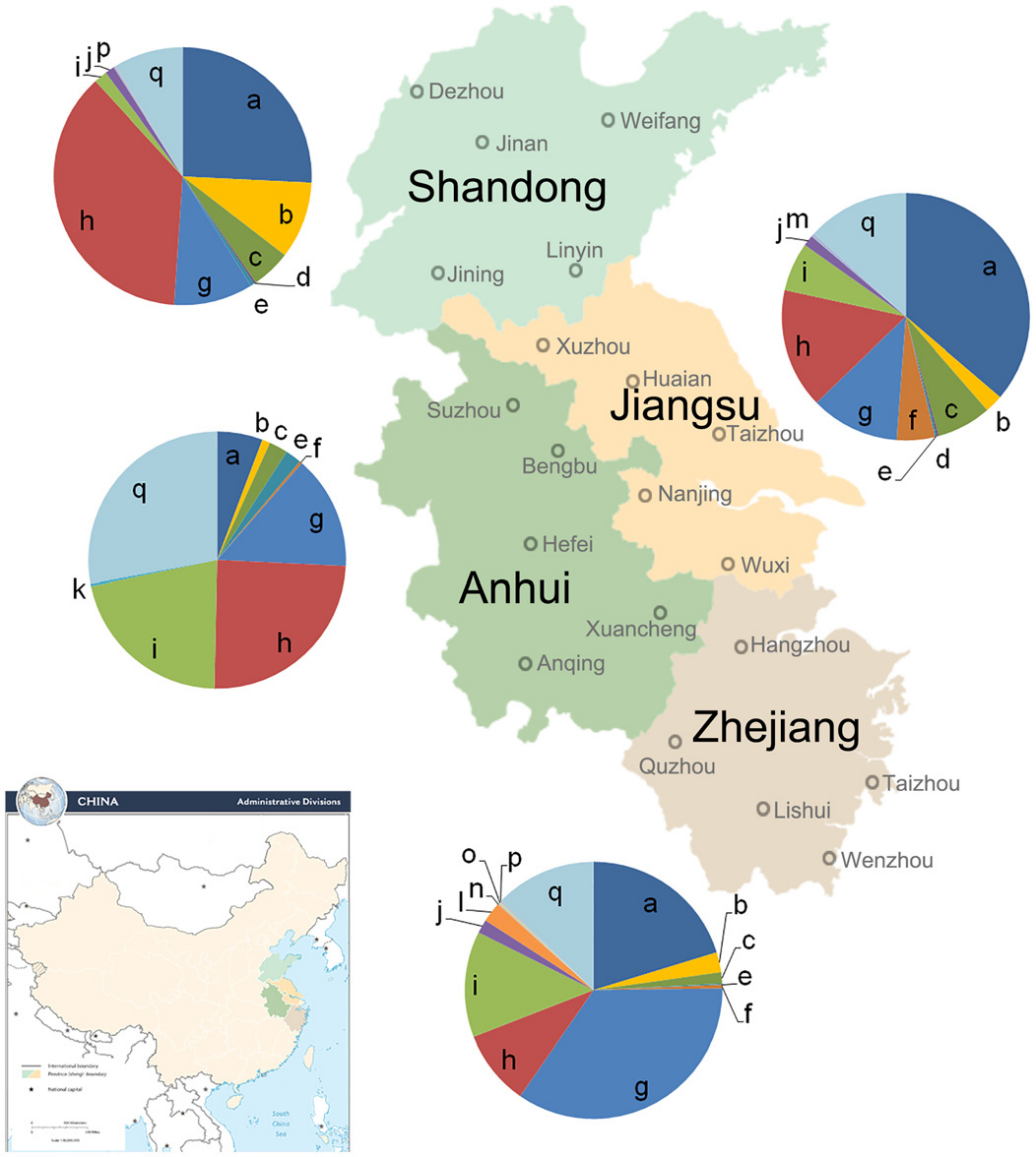
Distribution of *Trichoderma* species in East China. Individual species are abbreviated as follows: a—*T*. *asperellum*, b—*T*. *atroviride*, c—*T*. *brevicompactum*, d—*T*. *capillare*, e—*T*. *erinaceum*, f—*T*. *fertile*, g—*T*. *hamatum*, h—*T*. *harzianum*, i—*T*. *koningiopsis*, j—*T*. *longibrachiatum*, k—*T*. *oblongisporum*, l—*T*. *pleuroticola*, m—*T*. *polysporum*, n—*T*. *saturnisporum*, o—*T*. *spirale*, p—*T*. *velutinum*, and q—*T*. *virens*.

A total of 14 species were identified from Zhejiang Province: *T*. *asperellum*, *T*. *atroviride*, *T*. *brevicompactum*, *T*. *erinaceum*, *T*. *fertile*, *T*. *hamatum*, *T*. *harzianum*, *T*. *saturnisporum*, *T*. *koningiopsis*, *T*. *pleuroticola*, *T*. *spirale*, *T*. *virens*, *T*. *longibrachiatum*, and *T*. *velutinum* (Fig 2); Among these, *T*. *asperellum*, *T*. *hamatum*, *T*. *koningiopsis*, and *T*. *virens* were the dominant species with dominance (*Y*) values of 0.086, 0.217, 0.025, and 0.045, respectively. *Trichoderma* isolates from Jiangsu Province comprised 12 species: *T*. *asperellum*, *T*. *atroviride*, *T*. *brevicompactum*, *T*. *erinaceum*, *T*. *fertile*, *T*. *hamatum*, *T*. *harzianum*, *T*. *koningiopsis*, *T*. *polysporum*, *T*. *virens*, *T*. *longibrachiatum*, and *T*. *capillare* (Fig 2); *T*. *asperellum*, *T*. *hamatum*, *T*. *harzianum*, and *T*. *virens* were dominant with dominance (*Y*) values of 0.159, 0.025, 0.05, and 0.029, respectively. *T*. *asperellum*, and *T*. *harzianum* were the dominant species with dominance (*Y*) values of 0.091 and 0.219, respectively, in 11 species that were found in Shandong Province; Other detected species include *T*. *atroviride*, *T*. *brevicompactum*, *T*. *capillare*, *T*. *erinaceum*, *T*. *hamatum*, *T*. *koningiopsis*, *T*. *longibrachiatum*, *T*. *velutinum*, and *T*. *virens* (Fig 2). *T*. *virens*, *T*. *hamatum*, *T*. *harzianum*, and *T*. *koningiopsis* were the dominant species with dominance (*Y*) values of 0.130, 0.038, 0.102, and 0.086, respectively, in the 10 species that were found in Anhui. Other detected species include *T*. *asperellum*, *T*. *atroviride*, *T*. *brevicompactum*, *T*. *erinaceum*, *T*. *fertile*, and *T*. *oblongisporum* (Fig 2).

**Fig 3:**
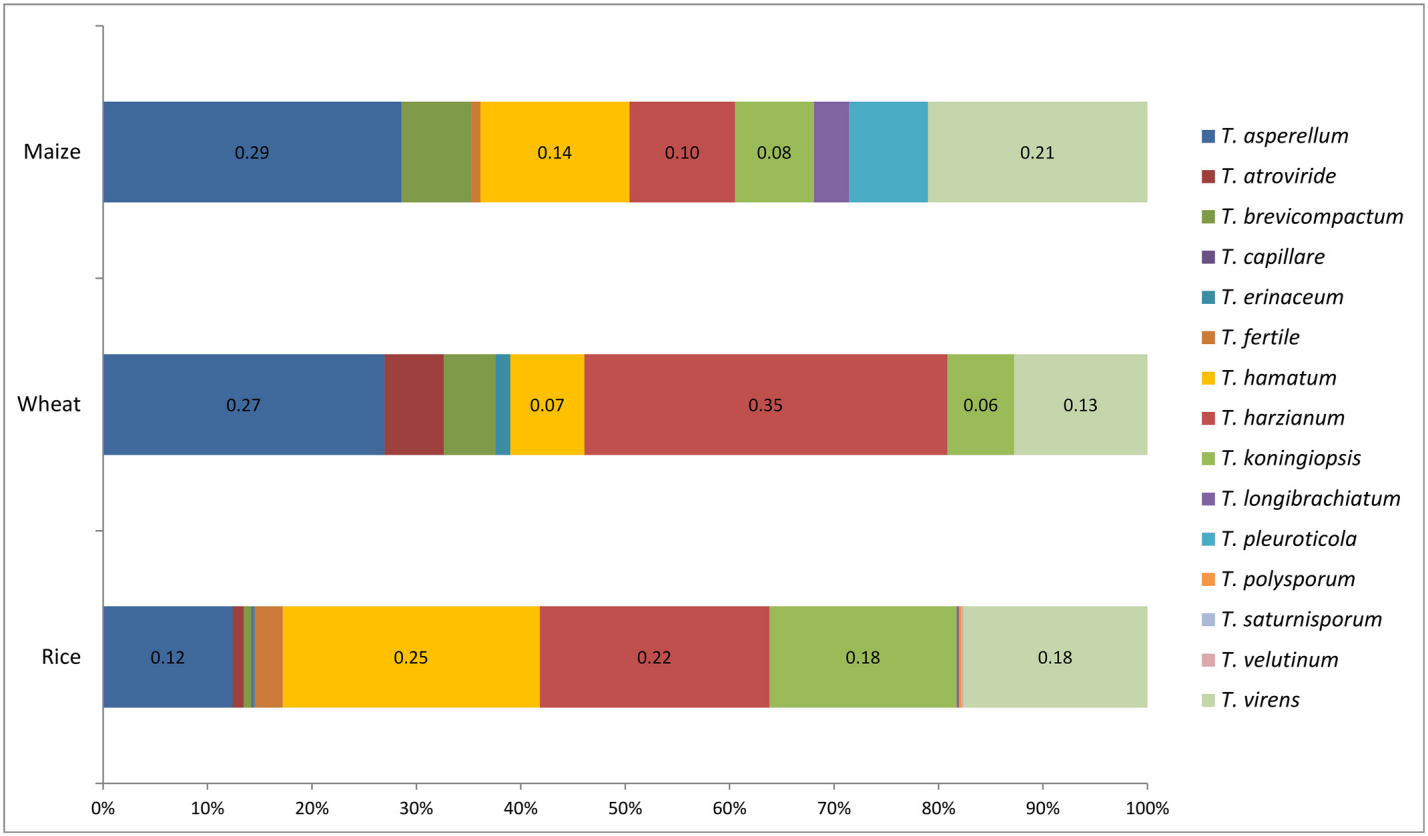
Relative frequencies of *Trichoderma* species isolated from rice, wheat, and maize soils. The relative frequencies of the main *Trichoderma* species are shown at the center of the bar.

There were significant differences in community of *Trichoderma* species from soils under different crop types (Fig 3). Rice soil yielded the highest species numbers, with 14 species being isolated from 942 strains. A total of nine and eight species were separated from maize and wheat soils, respectively. In addition, different *Trichoderma* species showed different relative frequencies in soils under different crop cultivation. The relative frequencies of *T*. *asperellum* ranged from 12 to 29%, and *T*. *harzianum* from 10 to 35%. The relative frequencies of *T*. *virens* exceeded 13% in all three cultivation types. We observed that relative frequencies of *T*. *hamatum* and *T*. *koningiopsis* under rice soil were higher than those under wheat and maize soil. Furthermore, the noticeable discrepancies indicate the preference of the *Trichoderma* to the crop soils.

**Fig 4:**
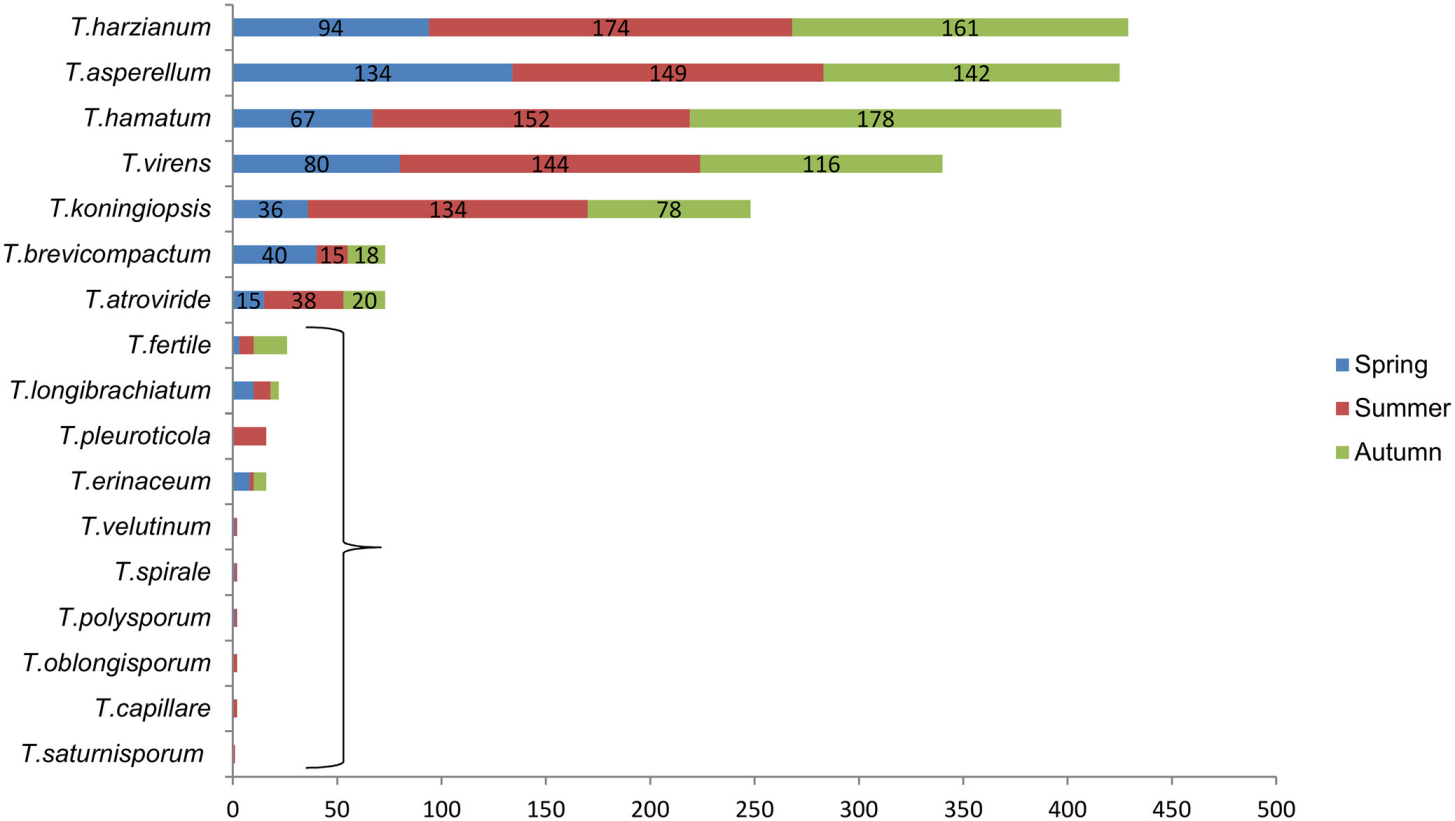
Distribution of *Trichoderma* species in different seasons. Inside box indicates the distribution of *T*. *fertile*, *T*. *longibrachiatum*, *T*. *pleuroticola*, *T*. *erinaceum*, *T*. *velutinum*, *T*. *spirale*, *T*. *polysporum*, *T*. *oblongisporum*, *T*. *saturnisporum*, and *T*. *capillare*. The numbers written on the bar charts correspond to the number of isolates per season.

The 2078 *Trichoderma* strains were collected over three seasons and the remarkable seasonal differences are shown in Fig 4. The amount of *Trichoderma* isolated during spring was clearly lower than during summer and autumn. For instance, the amounts of *T*. *virens*, *T*. *koningiopsis*, *T*. *hamatum* and *T*. *harzianum* in spring were lower than the amounts in summer and autumn. In terms of the number of diverse species, 17 species were observed in summer, followed by 13 in spring and 9 in autumn. *T*. *capillare*, *T*. *saturnisporum*, *T*. *oblongisporm*, and *T*. *pleuroticola* were only found in summer.

### Distribution of the dominant species T. hamatum in East China

There were 397 *Trichoderma* isolates, belonging to seven haplotypes, identified as *T*. *hamatum*. Haplotypes ham1 (337 isolates) and ham3 (44 isolates) accounted for the vast majority of isolates. Haplotypes ham4, ham5, and ham6 were monotypic. Five *T*. *hamatum* haplotypes were distributed in Zhejiang Province, which was the southernmost province in the investigated area. In addition, only haplotype ham1, which was the most distributed, was found in Shandong Province (the northernmost province in the investigated area). Furthermore, the number of *T*. *hamatum* isolates in Shandong Province was 42, much less than in Zhejiang Province (222 isolates) and Anhui Province (84).

**Fig 5:**
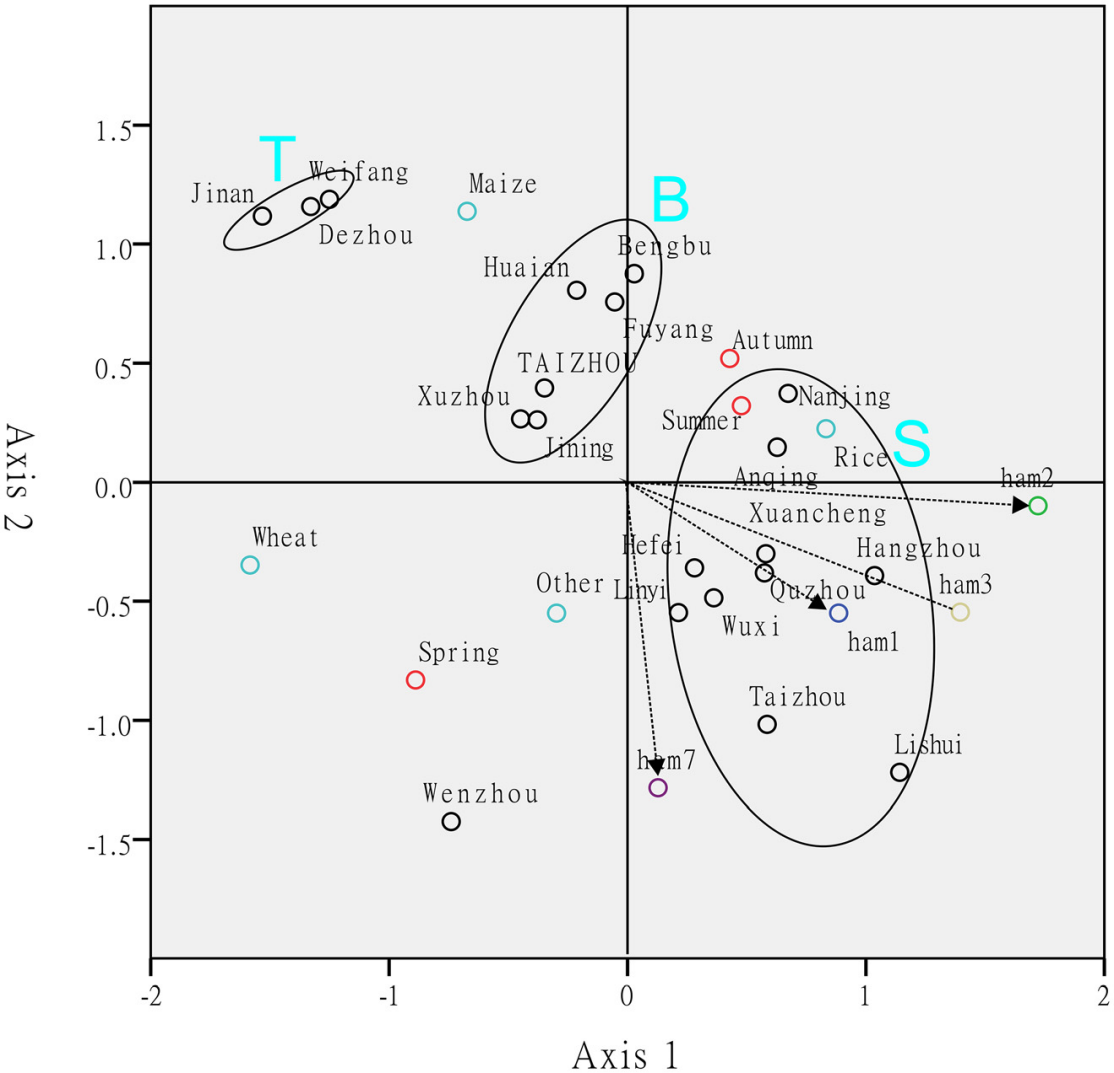
Multiple correspondence analysis of sampling sites based on the existence of *T*. *hamatum*. The inertia of the first and second axes is 0.33 and 0.28, respectively. Ellipsoids show the centre of the regions. S is the region of subtropical monsoon climate, T is the region of temperate monsoon climate, and B is the boundary region between the subtropical monsoon and the temperate monsoon climatic regions. Red circles indicate the seasons and cyan circles indicate the crop types. Other colored circles indicate the main haplotypes of *T*. *hamatum*.

The distribution of *T*. *hamatum* was indicated by indirect correspondence analysis (CA) based on the existence of *T*. *hamatum*. In CA bioplot, the closer two points appear, the closer their profile patterns are to each other. Fig 5 shows that Jinan, Weifang, and Dezhou, which belong to the temperate monsoon climate (T), were clustered together. Nanjing, Wuxi, Anqing, Quzhou, Taizhou, Linyi, Hangzhou, Hefei, Xuancheng, and Lishui formed an ellipsoid, and all belong to the lower latitude regions and subtropical monsoon climate (S), except for Linyi. Bengbu, Fuyang, Huaian, Xuzhou, Jining, and Taizhou, which clustered in an ellipsoid (B), were near the boundary between the subtropical monsoon climatic region and the temperate monsoon climatic region. Ellipsoid S was closer to main haplotypes of *T*. *hamatum* than Ellipsoids T and B, which indicates a strong correlation between Ellipsoid S and the main haplotypes of *T*. *hamatum*. When the magnitudes to haplotypes of *T*. *hamatum* were drawn, we found that the regions (ellipsoids) are arranged on a gradient from south (subtropical monsoon climate) to north (temperate monsoon climate) along the axis. Furthermore, in the bioplots of other dominant species (*T*. *virens*, *T*. *asperellum*, and *T*. *harzianum*), this distribution pattern was not seen. According to the distance between points in the bioplot (Fig 5), different crops yielded different *T*. *hamatum* profile patterns. Furthermore, summer and autumn present a similar *T*. *hamatum* profile pattern, whereas that of spring is different.

## Discussion

Morphological analysis is a poor method for *Trichoderma* species identification because of character plasticity [32]. Therefore, all putative *Trichoderma* isolates in this study were identified from selective medium. ITS rDNA sequences were also submitted to the oligonucleotide barcode program *TrichO*KEY [30] for identification. The accuracy and convenience of this method has enabled the completion of many studies over large regions such as China [21, 23], Colombia [22, 33], and Europe [7, 16, 34–36]. Although similar research has been completed, noticeable differences were still found. Additional details of *Trichoderma* biodiversity appear in our study.

In our study, the composition and proportion of *Trichoderma* species and the dominant species were distinct between different regions and cropping types. Zhejiang Province had 14 species and the most dominant species in Zhejiang Province was *T*. *hamatum*, followed by Jiangsu (12, *T*. *asperellum*), Shandong (11, *T*. *harzianum*) and Anhui (10, *T*. *virens*). The Shannon–Wiener diversity index of haplotypes also suggested that Zhejiang Province has the greatest level of *Trichoderma* diversity. Environmental preference of *Trichoderma* has also been found in many studies [37–40].

Significant differences of *Trichoderma* communities in different crop soil were observed in our study. Variation in the amount and chemical composition of the plant root exudation may contribute to the significant differences of *Trichoderma* communities. These plant root exudation, including carbohydrates, amino acids, organic acids, secondary metabolite and signaling molecules, can alter physico-chemical properties of soil, provide carbon and nitrogen sources and interacting with *Trichoderma* [41]. As *Trichoderma* have been considered as plant symbionts, the plant root may show a stronger influence to *Trichoderma*. The remarkable effects of plant root exudation on bacteria and phytopathogenic fungi communities have been observed in other studies [42]. Thus, we have reason to consider that the root exudation may be responsible for plant types-specific *Trichoderma* communities. In our study, the diversity of *Trichoderma* in rice soil is higher than wheat and corn. This may not only be attributed to the more beneficial exudation that rice roots exudates, but may also be associated with the soil moisture, pH, oxygen content and particle morphology that notably affected by cultivation measures in rice growth period. For example, we observed the preference of *T*. *hamatum* and *T*. *koningiopsis* to rice soil.

We found that fungal diversity was influenced by seasonal effects, supporting previous studies [19]. In our study, the amount and composition of *Trichoderma* were diverse in different seasons. Nelson et al. showed that *T*. *hamatum* was more abundant in spring than in summer [43], which was contradictory to our results. This result may be attributed to differences in climate, soil depth or plant type. More likely, the misidentified *T*. *hamatum* accounts for a greater proportion since the concept of aggregate species was adopted at that time [44]. Of interest is that *T*. *asperellum* has few clear changes between seasons.

Compared with the report of Sun et al.[23], a similar diversity pattern with several species forming a co-dominant group was observed in soil samples from East China. In both sun et al. and our studies, *T*. *asperellum*, *T*. *virens*, *T*. *harzianum*, and *T*. *hamatum* were the most frequent species. However, *T*. *koningiopsis* was frequently isolated in our study while only one strain was found by Sun. *T*. *viride* was on an exception. These results may be related to the latitudinal difference of the sample sites. *T*. *koningiopsis* is essentially a tropical species [45] and *T*. *viride* is a representation of the temperate *Trichoderma* biota [46]. We found that uncommon species were different in proportion and composition; for example, *T*. *citrioviride* and *T*. *koningii* were not found in our study. Instead of focusing on the description of *Trichoderma* biodiversity, we explored the distribution pattern at the haplotype level and attempted to identify the factors.

Based on our study, *T*. *hamatum* was the dominant species in farmland soil. The dominance of *T*. *hamatum* in farmland soil may be related to higher stress-tolerance and excellent competition at soil temperatures of 20–25°C [47–49]. We also found that the south to north distribution of *T*. *hamatum*, which was consistent with the change of climate types. The decreasing precipitation from south to north is the main feature of East China. The increased tolerance of *T*. *hamatum* to excessive moisture above other species may contribute to this situation.

*T*. *harzianum* has a worldwide distribution and is often the dominant species in other environments, such as Egypt [50, 51], Asia [39] and Europe [34]. As the most dominant species in our study, *T*. *harzianum* constituted of seven haplotypes, which were strongly supported by the phylogenetic tree. High genetic diversity may contribute to the higher abundance of *T*. *harzianum*. Haplotypes harz1 and asper1 are able to adapt well to a varied environment and are dominant species, so they are potentially important agents for use in biological control.

A level of *Trichoderma* biodiversity in agricultural soil was reported in this study. *Trichoderma* biodiversity in agricultural fields varies with season, region, and crop type. Of the four dominant species identified, only *T*. *hamatum* showed a south to north distribution. However, some species which grow slowly or sporulate later in the year may not have been isolated due to the limitations of the cultivation-dependent methods that we used. A combination of cultivation-dependent and cultivation-independent methods should be used to provide further information in future exploration of *Trichoderma* biodiversity in agricultural soils.

## Supporting Information

S1 Fig Comparison of ITS sequences from the representative strains of haplotypes of T. asperellum and type strain *T*. *asperellum* CBS 433.97. At the position of 115 and 371, base deletion or transversion was observed in asper1, 4 and 7.
(TIF)

S1 Table Geographical coordinates of the sampling sites. (DOCX)
