# Supplementary figures and images for: Trichoderma Biodiversity of Agricultural Fields in East China Reveals a Gradient Distribution of Species
Source: PLoS One. 2016 Aug 2;11(8):e0160613. doi: 10.1371/journal.pone.0160613 (PMC4970770; doi:10.1371/journal.pone.0160613)

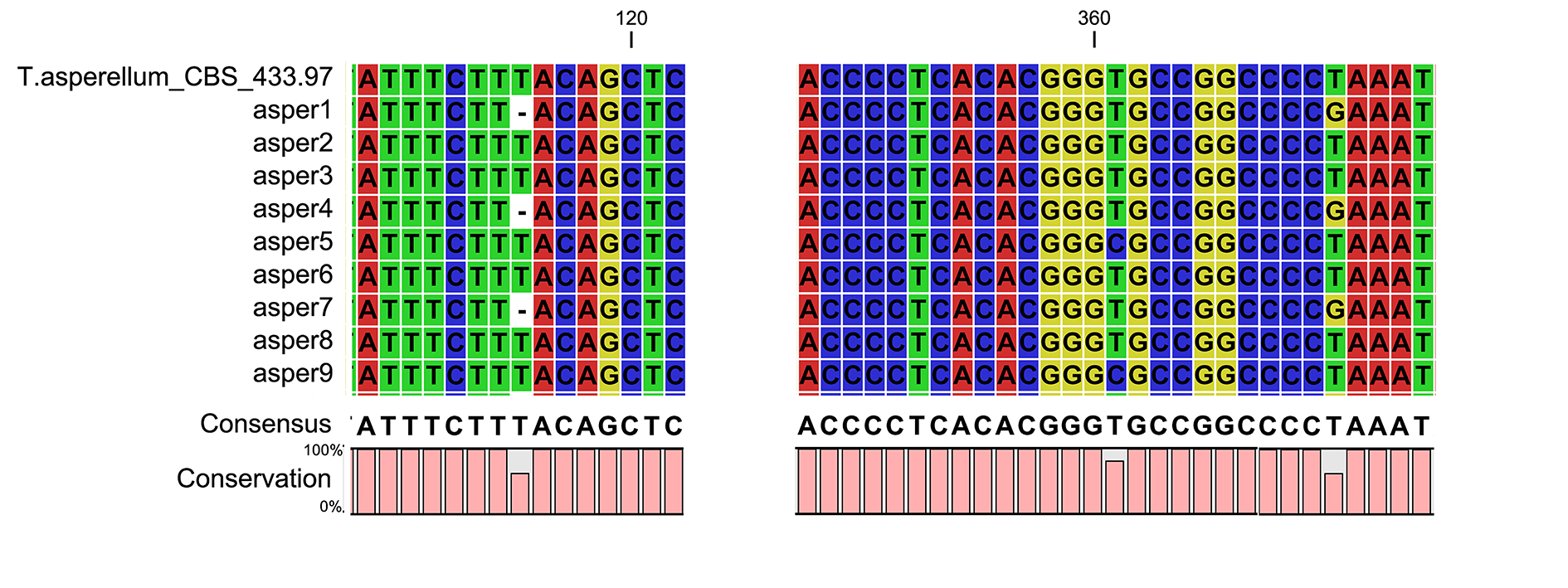

Supplement: S1 Fig — At the position of 115 and 371, base deletion or transversion was observed in asper1, 4 and 7. (TIF) [file pone.0160613.s001.tif]
