# Supplementary material for: Trichoderma Biodiversity of Agricultural Fields in East China Reveals a Gradient Distribution of Species
Source: PLoS One. 2016 Aug 2;11(8):e0160613. doi: 10.1371/journal.pone.0160613 (PMC4970770; doi:10.1371/journal.pone.0160613)
Supplement: S1 Table — (DOCX) [file pone.0160613.s002.docx]

**S1 Table. Geographical coordinates of the sampling sites**

| **Province** | **City** | **Latitude** | **Longitude** |
| --- | --- | --- | --- |
| **Jiangsu** | Xuzhou | N 34°04'47.5" | E 117°12'13.5" |
|  |  | N 34°04'49.9" | E 117°12'14.0" |
|  |  | N 34°04'49.9" | E 117°12'14.0" |
|  |  | N 34°04'46.8" | E 117°12'13.9" |
|  |  | N 34°04'45.1" | E 117°12'13.5" |
|  |  | N 34°04'46.8" | E 117°12'13.9" |
|  |  | N 34°04'31.9" | E 117°12'07.3" |
|  |  | N 34°04'32.1" | E 117°12'05.3" |
|  |  | N 34°04'32.4" | E 117°12'01.7" |
|  |  | N 34°10'15.4" | E 117°20'37.6" |
|  |  | N 34°10'45.5" | E 117°20'48.2" |
|  |  | N 34°05'27.8" | E 117°11'54.5" |
|  | Taizhou | N 32°29'26.0" | E 120°06'33.0" |
|  |  | N 32°29'27.9" | E 120°06'32.4" |
|  |  | N 32°29'27.0" | E 120°06'33.6" |
|  |  | N 32°32'50.6" | E 120°05'30.6" |
|  |  | N 32°32'48.5" | E 120°05'31.4" |
|  |  | N 32°32'50.5" | E 120°05'28.8" |
|  |  | N 32°32'26.9" | E 120°06'11.2" |
|  |  | N 32°32'26.8" | E 120°06'10.0" |
|  |  | N 32°32'27.0" | E 120°06'09.4" |
|  |  | N 32°32'51.6" | E 120°05'59.4" |
|  |  | N 32°29'45.7" | E 120°06'45.5" |
|  |  | N 32°28'56.3" | E 120°07'05.5" |
|  | Huaian | N 33°38'42.7" | E 118°58'24.1" |
|  |  | N 33°38'42.0" | E 118°58'23.8" |
|  |  | N 33°38'41.2" | E 118°58'23.8" |
|  |  | N 33°38'30.8" | E 118°56'22.0" |
|  |  | N 33°38'30.7" | E 118°56'21.1" |
|  |  | N 33°38'30.6" | E 118°56'20.4" |
|  |  | N 33°37'54.0" | E 118°56'11.6" |
|  |  | N 33°37'54.0" | E 118°56'10.2" |
|  |  | N 33°37'54.5" | E 118°56'06.8" |
|  |  | N 33°37'50.1" | E 118°56'17.8" |
|  |  | N 33°37'54.5" | E 118°56'06.8" |
|  |  | N 33°37'50.5" | E 118°56'15.9" |
|  | Nanjing | N 32°22'28.9" | E 118°49'47.2" |
|  |  | N 32°23'41.1" | E 118°49'42.3" |
|  |  | N 32°25'10.4" | E 118°51'14.1" |
|  |  | N 32°25'10.9" | E 118°51'13.2" |
|  |  | N 32°25'11.0" | E 118°51'11.8" |
|  |  | N 32°22'15.5" | E 118°48'52.5" |
|  |  | N 32°21'57.5" | E 118°48'54.6" |
|  |  | N 32°21'57.6" | E 118°48'55.3" |
|  |  | N 32°21'57.8" | E 118°48'55.4" |
|  |  | N 32°22'14.8" | E 118°48'54.1" |
|  |  | N 32°25'09.3" | E 118°51'14.7" |
|  |  | N 32°22'16.3" | E 118°48'40.7" |
|  | Wuxi | N 31°23'31.2" | E 119°53'04.3" |
|  |  | N 31°23'30.2" | E 119°53'05.3" |
|  |  | N 31°23'29.7" | E 119°53'05.2" |
|  |  | N 31°22'16.5" | E 119°56'56.5" |
|  |  | N 31°22'17.2" | E 119°56'55.7" |
|  |  | N 31°22'17.3" | E 119°56'54.5" |
|  |  | N 31°22'09.8" | E 119°55'57.5" |
|  |  | N 31°23'03.4" | E 119°54'07.6" |
|  |  | N 31°23'03.1" | E 119°54'07.0" |
|  |  | N 31°21'55.3" | E 119°56'47.6" |
|  |  | N 31°22'09.8" | E 119°55'57.5" |
|  |  | N 31°22'24.3" | E 119°57'08.4" |
| **Anhui** | Anqing | N 30°29'54" | E 116°58'35" |
|  |  | N 30°29'58" | E 116°58'31" |
|  |  | N 30°29'50" | E 116°58'34" |
|  |  | N 30°28'54" | E 116°58'40" |
|  |  | N 30°28'44" | E 116°58'53" |
|  |  | N 30°28'49" | E 116°58'35" |
|  |  | N 30°26'33" | E 116°51'03" |
|  |  | N 30°26'35" | E 116°51'03" |
|  |  | N 30°26'40" | E 116°51'03" |
|  |  | N 30°25'42" | E 116°51'45" |
|  |  | N 30°25'25" | E 116°50'30" |
|  |  | N 30°25'2" | E 116°49'35" |
|  | Fuyang | N 32°40'07" | E 115°35'28" |
|  |  | N 32°40'10" | E 115°35'17" |
|  |  | N 32°40'01" | E 115°35'39" |
|  |  | N 32°43'00" | E 115°33'22" |
|  |  | N 32°40'14" | E 115°35'20" |
|  |  | N 32°40'15" | E 115°35'31" |
|  |  | N 32°39'20" | E 115°30'20" |
|  |  | N 32°39'30" | E 115°30'19" |
|  |  | N 32°39'36" | E 115°30'15" |
|  |  | N 32°46'11" | E 115°25'00" |
|  |  | N 32°42'00" | E 115°35'50" |
|  |  | N 32°40'08" | E 115°35'45" |
|  | Xuancheng | N 31°1'25" | E 118°51'31" |
|  |  | N 31°1'31" | E 118°51'38" |
|  |  | N 31°1'35" | E 118°51'32" |
|  |  | N 31°1'21" | E 118°0'50" |
|  |  | N 31°1'24" | E 118°0'59" |
|  |  | N 31°1'29" | E 118°0'54" |
|  |  | N 31°57'38" | E 118°50'49" |
|  |  | N 31°57'45" | E 118°50'59" |
|  |  | N 31°57'34" | E 118°50'41" |
|  |  | N 31°1'40" | E 118°5'17" |
|  |  | N 31°1'32" | E 118°4'59" |
|  |  | N 31°2'49" | E 118°5'59" |
|  | Hefei | N 31°39'42" | E 117°11'42" |
|  |  | N 31°39'49" | E 117°11'48" |
|  |  | N 31°39'45" | E 117°11'44" |
|  |  | N 31°37'1" | E 117°11'30" |
|  |  | N 31°37'8" | E 117°11'37" |
|  |  | N 31°37'17" | E 117°11'35" |
|  |  | N 31°38'10" | E 117°11'32" |
|  |  | N 31°38'2" | E 117°11'39" |
|  |  | N 31°38'0" | E 117°11'32" |
|  |  | N 31°36'47" | E 117°13'7" |
|  |  | N 31°36'11" | E 117°13'30" |
|  |  | N 31°35'9" | E 117°14'7" |
|  | Bengbu | N 32°56'37" | E 117°09'27" |
|  |  | N 32°56'47" | E 117°09'17" |
|  |  | N 32°56'41" | E 117°09'23" |
|  |  | N 32°54'51" | E 117°5'28" |
|  |  | N 32°54'55" | E 117°5'23" |
|  |  | N 32°54'59" | E 117°5'25" |
|  |  | N 33°2'21" | E 116°57'22" |
|  |  | N 33°2'23" | E 116°57'32" |
|  |  | N 33°2'27" | E 116°57'26" |
|  |  | N 33°2'34" | E 117°8'33" |
|  |  | N 32°56'55" | E 117°13'33" |
|  |  | N 32°57'38" | E 117°10'44" |
| **Zhejiang** | Wenzhou | N 28°19'5.98" | E 120°44'58.3" |
|  |  | N 28°19'5.48" | E 120°44'57.6" |
|  |  | N 28°19'4.98" | E 120°44'58.5" |
|  |  | N 28°19'6.92" | E 120°44'58.0" |
|  |  | N 28°19'4.93" | E 120°44'58.9" |
|  |  | N 28°19'5.95" | E 120°44'57.8" |
|  |  | N 28°19'4.94" | E 120°44'58.2" |
|  |  | N 28°19'6.99" | E 120°44'57.5" |
|  |  | N 28°19'4.91" | E 120°44'58.3" |
|  |  | N 28°18.903' | E 120°44.931' |
|  |  | N 27°59.150' | E 120°33.309' |
|  |  | N 28°18.903' | E 120°44.931' |
|  | Lishui | N 28°34'02.5" | E 119°12'02.7" |
|  |  | N 28°34'18.7" | E 119°12'22.4" |
|  |  | N 28°34'22.3" | E 119°12'15.8" |
|  |  | N 28°33'33.8" | E 119°10'15.4" |
|  |  | N 28°33'35.7" | E 119°10'06.5" |
|  |  | N 28°33'37.3" | E 119°10'09.7" |
|  |  | N 28°32'43.1" | E 119°10'12.8" |
|  |  | N 28°32'47.0" | E 119°10'09.6" |
|  |  | N 28°32'49.5" | E 119°10'08.3" |
|  |  | N 28°34'20.3" | E 119°12'20.8" |
|  |  | N 28°32'46.9" | E 119°10'04.0" |
|  |  | N 28°32'46.8" | E 119°10'04.9" |
|  | Taizhou | N 28°42'38.2" | E 121°26'57.5" |
|  |  | N 28°42'37.8" | E 121°26'56.2" |
|  |  | N 28°42'37.4" | E 121°26'55.1" |
|  |  | N 28°43'36.1" | E 121°25'24.6" |
|  |  | N 28°43'36.0" | E 121°25'23.3" |
|  |  | N 28°43'35.8" | E 121°25'23.2" |
|  |  | N 28°38'44.4" | E 121°21'46.9" |
|  |  | N 28°38'44.5" | E 121°21'46.3" |
|  |  | N 28°38'44.3" | E 121°21'47.8" |
|  |  | N 28°42'20.8" | E 121°28'43.5" |
|  |  | N 28°42'16.4" | E 121°28'47.1" |
|  |  | N 28°42'16.4" | E 121°28'49.8" |
|  | Hangzhou | N 29°18'43.2" | E 119°07'04.2" |
|  |  | N 29°18'45.1" | E 119°07'05.5" |
|  |  | N 29°18'40.0" | E 119°07'09.1" |
|  |  | N 29°12'42.1" | E 119°10'52.3" |
|  |  | N 29°12'44.5" | E 119°10'55.0" |
|  |  | N 29°12'45.9" | E 119°10'56.7" |
|  |  | N 29°19'24.6" | E 119°19'28.7" |
|  |  | N 29°19'27.7" | E 119°19'24.1" |
|  |  | N 29°19'27.1" | E 119°19'25.8" |
|  |  | N 29°32'52.3" | E 119°30'11.0" |
|  |  | N 29°32'54.5" | E 119°30'18.7" |
|  |  | N 29°33'16.9" | E 119°32'18.0" |
|  | Quzhou | N 28°37'37.1" | E 118°34'01.6" |
|  |  | N 28°37'36.9" | E 118°34'05.0" |
|  |  | N 28°37'36.8" | E 118°34'05.1" |
|  |  | N 28°37'41.8" | E 118°34'23.2" |
|  |  | N 28°37'41.0" | E 118°34'23.8" |
|  |  | N 28°37'40.4" | E 118°34'24.1" |
|  |  | N 28°39'36.2" | E 118°35'28.1" |
|  |  | N 28°39'37.0" | E 118°35'28.8" |
|  |  | N 28°39'35.9" | E 118°35'28.0" |
|  |  | N 28°37'32.2" | E 118°34'03.3" |
|  |  | N 28°39'36.8" | E 118°35'28.9" |
|  |  | N 28°37'34.5" | E 118°34'05.3" |
| **Shandong** | Jinan | N 36°32'25.2" | E 117°01'06.4" |
|  |  | N 36°32'28.3" | E 117°01'20.5" |
|  |  | N 36°32'28.4" | E 117°01'21.9" |
|  |  | N 36°33'26.5" | E 116°57'32.3" |
|  |  | N 36°33'40.4" | E 116°57'21.4" |
|  |  | N 36°33'39.6" | E 116°57'24.1" |
|  |  | N 36°33'39.9" | E 116°57'23.1" |
|  |  | N 36°33'46.3" | E 116°57'06.0" |
|  |  | N 36°33'45.7" | E 116°57'06.5" |
|  |  | N 36°32'33.3" | E 117°01'08.6" |
|  |  | N 36°29'12.8" | E 117°00'43.8" |
|  |  | N 36°29'31.6" | E 117°02'16.0" |
|  |  | N 36°33'44.7" | E 116°57'06.6" |
|  | Weifang | N 36°38'17.3" | E 119°02'21.3" |
|  |  | N 36°38'17.7" | E 119°02'22.1" |
|  |  | N 36°38'17.7" | E 119°02'23.0" |
|  |  | N 36°37'50.8" | E 119°01'32.2" |
|  |  | N 36°37'51.0" | E 119°01'32.9" |
|  |  | N 36°37'50.7" | E 119°01'32.4" |
|  |  | N 36°40'29.2" | E 119°00'15.0" |
|  |  | N 36°40'29.4" | E 119°00'15.5" |
|  |  | N 36°40'29.0" | E 119°00'16.6" |
|  |  | N 36°38'17.3" | E 119°02'21.3" |
|  |  | N 36°38'27.7" | E 119°01'21.5" |
|  |  | N 36°40'39.8" | E 119°00'07.5" |
|  | Dezhou | N 37°21'21.5" | E 116°21'06.7" |
|  |  | N 37°21'21.7" | E 116°21'04.2" |
|  |  | N 37°21'04.6" | E 116°21'04.6" |
|  |  | N 37°20'43.5" | E 116°23'08.2" |
|  |  | N 37°21'11.9" | E 116°22'53.5" |
|  |  | N 37°21'12.9" | E 116°22'54.1" |
|  |  | N 37°21'40.7" | E 116°25'35.3" |
|  |  | N 37°21'39.8" | E 116°25'34.3" |
|  |  | N 37°21'41.1" | E 116°25'33.9" |
|  |  | N 37°22'08.6" | E 116°19'37.4" |
|  |  | N 37°21'58.4" | E 116°20'09.8" |
|  |  | N 37°21'07.6" | E 116°23'40.3" |
|  | Linyi | N 35°11'28.9" | E 118°28'36.0" |
|  |  | N 35°11'29.4" | E 118°28'37.0" |
|  |  | N 35°11'30.1" | E 118°28'37.1" |
|  |  | N 35°11'19.3" | E 118°29'49.1" |
|  |  | N 35°11'18.6" | E 118°29'50.1" |
|  |  | N 35°11'19.1" | E 118°29'48.0" |
|  |  | N 35°08'22.5" | E 118°30'24.9" |
|  |  | N 35°08'20.9" | E 118°30'25.1" |
|  |  | N 35°08'22.4" | E 118°30'26.0" |
|  |  | N 35°11'12.1" | E 118°26'28.6" |
|  |  | N 35°09'53.2" | E 118°31'28.6" |
|  |  | N 35°11'42.1" | E 118°27'24.8" |
|  | Jining | N 34°56'03.8" | E 116°30'19.1" |
|  |  | N 34°56'03.8" | E 116°30'19.1" |
|  |  | N 34°56'03.3" | E 116°30'17.3" |
|  |  | N 34°57'40.2" | E 116°32'37.6" |
|  |  | N 34°57'40.8" | E 116°32'36.9" |
|  |  | N 34°57'41.2" | E 116°32'38.1" |
|  |  | N 34°58'24.9" | E 116°34'59.6" |
|  |  | N 34°58'23.7" | E 116°35'00.0" |
|  |  | N 34°58'23.0" | E 116°34'59.5" |
|  |  | N 34°56'21.2" | E 116°29'03.3" |
|  |  | N 34°56'47.5" | E 116°28'39.3" |
|  |  | N 34°56'45.7" | E 116°28'24.3" |
